# Association of Macrophage Migration Inhibitory Factor Polymorphisms with Total Plasma IgE Levels in Patients with Atopic Dermatitis in Korea

**DOI:** 10.1371/journal.pone.0162477

**Published:** 2016-09-06

**Authors:** Jung soo Kim, Jinyoung Choi, Hyung-Jin Hahn, Young-Bok Lee, Dong-Soo Yu, Jin-Wou Kim

**Affiliations:** Departments of Dermatology, College of Medicine, The Catholic University of Korea, Uijongbu-City, Kyonggi-do, Korea; Ohio State University, UNITED STATES

## Abstract

The macrophage migration inhibitory factor (*MIF*) gene is located on human chromosome 22q11.2 and is linked to atopic phenotypes. Plasma MIF and log [total IgE] levels are significantly elevated in atopic dermatitis (AD) patients. The aim of this study was to evaluate the relationship between two *MIF* polymorphisms, −173 G to C and −794 CATT_5–8_, and total plasma IgE levels in AD patients in Korea. We performed PCR-RFLP analysis in 178 AD patients and 80 control subjects to determine whether *MIF* SNPs are associated with susceptibility to AD. Plasma total IgE and MIF levels were determined, and then logistic regression analyses were performed to determine the associations between a SNP or haplotype and plasma total IgE or MIF levels. The −173 G/C polymorphism, located in the *MIF* promoter, was significantly associated with AD; the odds ratios (ORs) for the CC homozygotes and GC heterozygotes were 9.3 and 2.5, respectively. The *MIF* C/5-CATT and the *MIF* C/7-CATT haplotypes were significantly associated with AD; the ORs for the *MIF* C/5-CATT and *MIF* C/7-CATT haplotypes were 9.7 and 4.5, respectively. Log [total IgE] levels were highly associated with the *MIF* −794 7-CATT polymorphism. Notably, the *MIF* C/7-CATT haplotype was associated with a decrease in plasma log [total IgE] levels in a gene dose-dependent manner. Although log [MIF] levels were not associated with the *MIF* polymorphisms, the frequencies of the *MIF* C/5-CATT haplotype-containing genotypes decreased in order of MIF levels. Our results demonstrate that *MIF* promoter polymorphisms in the −173 C allele and the *MIF* C/5-CATT and C/7-CATT haplotypes were significantly associated with an increased risk for AD. In particular, the −794 7-CATT locus and the *MIF* C/7-CATT haplotype were significantly associated with decreased total IgE levels in the plasma, suggesting that these polymorphisms might be a marker for intrinsic AD rather than extrinsic AD that shows high total IgE levels and presence of allergen-specific IgE.

## Introduction

Atopic dermatitis (AD) is a multifactorial skin disease that appears to be affected by both genetic and environmental factors [1, 2]. Macrophage migration inhibitory factor (MIF) is a proinflammatory cytokine [3], and serum MIF levels and regional skin lesions increase significantly in AD patients [4, 5]. Peripheral blood mononuclear cells are an important source of increased serum MIF in AD patients [6]. In addition, a subpopulation of AD patients has pollen-induced allergic conjunctivitis or pollen dermatitis, in which MIF levels are increased, leading to the accumulation of eosinophils in the conjunctiva and eyelid dermis [7].

The *MIF* gene maps to human chromosome 22q11.2 [8]. Polymorphisms in the *MIF* promoter region are reported to have a functional relationship with AD; a single nucleotide polymorphism (SNP), −173 G to C (rs755622) [9, 10], and a tetranucleotide CATT repeat, beginning at nucleotide position −794 (rs5844572) [11], are associated with altered MIF expression levels. The *MIF* −173 C allele also confers an increased susceptibility to AD and higher MIF protein expression [10]. These polymorphisms in the *MIF* gene are also associated with several immune-mediated inflammatory diseases, including atopy [12], asthma [13], juvenile idiopathic arthritis [9], rheumatoid arthritis[11], psoriasis [14, 15], and psoriatic arthritis [10, 16], suggesting that the polymorphisms are functionally important.

MIF, first detected in the supernatants from T lymphocyte cultures, was found to have immune activity [5] and to be involved in macrophage activation and antigen-driven T cell responses [17]. In addition, MIF regulates innate immune responses through the modulation of Toll-like receptor 4 (TLR4) in macrophages [18]. Plasma MIF concentration is significantly higher in patients with extrinsic AD than in those with intrinsic AD [19]. In addition, plasma MIF concentrations in AD patients are positively correlated with the *Dermatophagoides farinae* (*Df*)-specific IgE score.

The plasma log [total IgE] levels also significantly increased in AD patients when compared to the levels in control subjects. Allergic, or extrinsic, AD is the classical type, with high prevalence and a rather poor prognosis, whereas nonallergic, or intrinsic AD, represents approximately 20% of incidence and is predominantly found in females [1, 20, 21]. Extrinsic AD increases plasma total IgE and specific IgE levels for environmental and food allergens. In contrast, intrinsic AD does not elevate total IgE or specific IgE levels.

Although MIF and plasma total IgE levels are associated with AD, little is known about the association between *MIF* promoter polymorphisms and plasma total IgE levels. In this study, we examined the association between two *MIF* polymorphisms, −173 G to C and −794 CATT_5–8_, and plasma total IgE levels in Korean AD patients.

## Materials and Methods

### Subjects

The study included 178 unrelated AD patients (95 males and 83 females; mean age 26.4 ± 14.5 years; range, 5–71 years) who were enrolled through the Department of Dermatology, The Catholic University Hospital in Korea. All patients had moderate to severe AD in accordance with the criteria of Hanifin and Rajka [22]. The control subjects included 80 healthy individuals without a personal or familial history of atopic diseases; all subjects were Korean. Blood was collected by venipuncture for the genetic studies, and genomic DNA was separated from the cell pellet using conventional methods (QIAamp blood kit, Qiagen, Hilden, Germany). Plasma total IgE was measured using the LPIA-200 system (Iatron Corp., Tokyo, Japan). The range of plasma IgE levels was 2–50,000 IU/mL (median [25^th^-75^th^ percentile]: 160.0 [51.5–813.0]). The Pharmacia CAP FEIA immunoassay was used to detect specific IgE antibodies to *D*. *pteronyssinus* (*Dp*) and *Df* on a UniCAP 100 automatic analyzer (Pharmacia and Upjohn; Uppsala, Sweden) in accordance with the manufacturer’s instructions. An antigen-specific IgE value > 0.35 kU/L was considered elevated.

### Ethics statement

This study was performed from Mar. 3rd 2003 to Dec. 25th 2004 in accordance with the principles of the Declaration of Helsinki and approved by the IRB in Uijongbu-City St. Mary's Hospital before the study began. Since there was no statutory law during that time, we obtained only verbal consent from the participants after explaining our study’s purpose and their rights. Whenever children/minors are included in the study, the parent/guardian were orally informed and agreed the purpose and procedure of our study. The specimen was discarded on Jan. 2005 based on the Bioethics and Safety Act, which administered at Jan. 1st 2005 in Korea. We just reanalyzed coded research data collected from Mar. 3rd 2003 to Dec. 25th 2004 under supervision of principle investigator and the Ethics Committee in Uijongbu-City St. Mary's Hospital re-approved this study at Nov. 12th 2015 (IRB: UC15RISI0161).

### Plasma MIF assay

Plasma MIF concentrations were measured using an enzyme-linked immunosorbent assay (ELISA) in accordance with the manufacturer’s instructions. A MIF monoclonal antibody (MAB 289; R&D Systems, Minneapolis, MN) against human MIF was coated onto the plate and a biotinylated MIF antibody was used for detection. The detection limit of the assay was 31.25 pg/mL.

### Identification of polymorphisms

Genomic DNA was amplified by polymerase chain reaction (PCR) to identify the CATT tetranucleotide repeat polymorphism beginning at position –794. The reactions were performed in 25 μL of reaction mixture containing 50 ng DNA, 250 μM dNTPs, 1.5 mM MgCl_2_, 10× buffer (Perkin-Elmer, Norwalk, CT, USA), 2.5 U *Taq* polymerase, and 20 pmol of the primers 5′-TGCAGGAACCAATACCCATAGG-3′ and 5′-GTCCCCGAGTTTACCATT-3′. The following reaction conditions were used: initial denaturation at 95°C for 12 min, followed by 35 cycles at 95°C for 30 s, 58°C for 30 s, and 72°C for 1 min, and then by a final extension step at 72°C for 10 min. The PCR products were separated by gel electrophoresis and visualized using the Silverstar Staining Kit (Bioneer, Daejeon, Korea). Allele sizes were determined in each subject using the LabWorks analysis program (UVP, Upland, CA, USA).

Genotyping of the –173 G/C polymorphism was performed by polymerase chain reaction-restriction fragment length polymorphism (PCR-RFLP). PCR was performed in a 25-μL mixture containing 50 ng DNA, 250 μM dNTPs, 1.5 mM MgCl_2_, 10× buffer, 2.5 U *Taq* polymerase, and 20 pmol of the primers 5′-ACTAAGAAAGACCCGAGGC-3′ and 5′-GGGGCACGTTGGTGTTTAC-3′. The following reaction conditions were used: initial denaturation at 95°C for 5 min, followed by 30 cycles at 95°C for 45 s, 60°C for 45 s, and 72°C for 45 s, and then by a final extension step at 72°C for 5 min. PCR-amplified DNA was digested with *Alu*I. Products were visualized by electrophoresis on a 3% (w/v) Nusieve GTG agarose gel stained (Lonza Rockland, Inc., Rockland, ME, USA) with ethidium bromide [9].

### Statistics

Hardy–Weinberg equilibrium was analyzed using gene frequencies obtained by simple gene counting, and the chi-square test was used to compare observed and expected values. Haplotype frequencies were estimated using the Phase 2.0 program, as described previously, and inferred using a Bayesian approach incorporating *a priori* expectations of haplotype structure from population genetics and coalescent theory [23]. Phase probabilities for each site were calculated for each subject. The genetic effects of SNPS and of inferred haplotypes were analyzed in the same way. The chi-square test and student’s *t*-test were used to compare genotype and haplotype frequencies for each *MIF* polymorphism. Odds ratios (OR) and 95% confidence intervals (CIs) were calculated using SAS ver. 8.1 software (SAS Institute, Cary, NC, USA). *P* < 0.05 was considered significant. The OR provides an effect estimate, where scores < 1 are associated with a protective effect and scores > 1 are associated with an increased risk. The genotypic distribution of the *MIF* SNPs and haplotypes in AD patients and in control subjects were analyzed with logistic regression models adjusted for age and sex, with log-transformed plasma total IgE levels as a covariate.

## Results

### Analysis of the *MIF* −173 and −794 allele and genotype frequencies

The clinical characteristics of the 258 subjects are shown in Table 1. The mean age was older in the controls, and males predominated in both groups. About 60% of AD patients were positive for *Dp*-specific IgE, which was measured by a fluoro-enzyme immunoassay, and 61% of patients were *Df* positive. AD patients had higher plasma Log [total IgE] levels (Student’s *t*-test, *p* < 0.0001) and Log [MIF] levels than control subjects (Student’s *t*-test, *p* = 0.0096). No deviation from Hardy–Weinberg equilibrium was observed for either polymorphism in either group. The genotypic distributions of the *MIF* −173 and −794 polymorphisms in both groups are shown in Table 1. The *MIF* −173 genotypic frequencies were not different between the two groups (*p* = 0.126). Similarly, genotypic frequencies of the *MIF* CATT repeat element were not different between the groups (*p* = 0.845).

**Table 1:** Genotypic frequencies of *MIF* promoter polymorphisms in 258 Korean subjects.

		Atopic dermatitis (N = 178)	Controls (N = 80)	P*
Age in years:	Median (range)	22 (5–71)	45 (24–80)	<0.0001
Sex:	Female/Male	83/ 95	23/ 57	0.007
Positive rate of *Dp*-specific IgE (%)		60	UD	UD
Positive rate of *Df*-specific IgE (%)		61	UD	UD
Log [IgE] ± SD		5.51 ± 1.64*	4.16 ± 1.20	<0.0001
Log [MIF] ± SD		5.87 ± 1.58*	5.20 ± 2.03	0.0096
Genotype (%)				
-173 G/C	GG	117 (65.7)	61 (76.6)	0.126
	GC	51 (28.7)	18 (22.5)	
	CC	10 (5.6)	1 (1.3)	
-794 CATT repeat	5,5	32 (18.0)	15 (18.8)	0.845
	5,6	72 (40.5)	35 (43.8)	
	5,7	15 (8.4)	6 (7.5)	
	5,8	1 (0.6)	0 (0)	
	6,6	27 (15.2)	11 (13.8)	
	6,7	27 (15.2)	9 (11.3)	
	7,7	4 (2.3)	4 (5.0)	

* Student’s *t*-test was used where appropriate.

*MIF*: macrophage migration inhibitory factor, *Dp*: *Dermatophagoides pteronyssinus*, *Df*: *Dermatophagoides farina*, UD: undetectable.

A logistic regression analysis was performed after controlling for age and sex as co-variables in all three analytical models (co-dominant, dominant, and recessive models for a rare allele) to show alternative effects of the variants. Both *MIF* −173 G/C promoter polymorphisms were significantly associated with AD (Table 2); the OR for CC homozygotes of the *MIF* −173 G/C polymorphism was 9.3 (compared to GG homozygotes, 95% CI, 1.02–84.37; *p* = 0.048), and the OR for GC heterozygotes of the *MIF* −173 G/C polymorphism was 2.5 (compared to GG homozygotes, 95% CI, 1.09–5.55, *p* = 0.031). No significant differences were observed in the ORs for noncarriers of the *MIF* 5-CATT allele or for the *MIF* −794 [CATT] _5–8_ repeat polymorphism, when compared to *MIF* 5-CATT homozygotes in AD patients (Table 2).

**Table 2:** Impact of the *MIF* –173G/C and –794 [CATT]_5–8_ polymorphisms [OR (95% CI)] on atopic dermatitis.

AD		-173 G/C	ORs (95% CI)	-794 [CATT]_5–8_ repeat	ORs (95% CI)
	Adjustments	-173 G		5-CATT	
	None	+/+	1.0 (reference)	+/+	1.0 (reference)
		+/-	1.5 (0.80–2.75)	+/-	1.0 (0.49–2.06)
		-/-	5.2 (0.65–41.68)	-/-	1.1 (0.52–2.46)
	Age, sex,	+/+	1.0 (reference)	+/+	1.0 (reference)
	Log [total IgE] levels	+/-	2.5 (1.09–5.55)*	+/-	0.9 (0.34–2.19)
		-/-	9.3 (1.02–84.37)**	-/-	1.3 (0.48–3.46)

Matching factors and potential confounding factors were adjusted for the unconditional logistic-regression analysis.

The analysis for atopic dermatitis was adjusted for age, sex, and log-transformed total plasma IgE levels.

OR: odds ratio; CI: confidence interval.

* *P* = 0.031.

**P = 0.048.

### *MIF* haplotype analyses

We reconstructed haplotype frequencies for each possible haplotype based on the observed genotype data for the *MIF* −173 G/C and −794 CATT alleles. A logistic regression analysis was conducted with the co-dominant model after controlling for age and sex with log [total IgE] levels as a co-variable. When the *MIF* G/5-CATT was selected as the reference, the frequencies of the common haplotypes were significantly different between the two groups (Table 3).

**Table 3:** Impact of estimated haplotype frequencies of the *MIF –*173 G/C and –794 CATT repeat polymorphisms.

Haplotype	Atopic dermatitis (%)	Controls (%)	*P*^*a*^	OR (95%CI)
G/5-CATT	139 (39.0)	68 (42.5)		1.0 (reference)
G/6-CATT	130 (36.5)	57 (35.6)	0.330	1.3 (0.47–3.44)
G/7-CATT	16 (4.5)	15 (9.4)	0.203	0.5 (0.20–1.40)
**C/5-CATT**	**13 (3.7)**	**3 (1.9)**	**0.009**	**9.7 (1.78–52.80)**
C/6-CATT	23 (6.5)	9 (5.6)	0.643	1.3 (0.47–3.44)
**C/7-CATT**	**34 (9.6)**	**8 (5.0)**	**0.005**	**4.5 (1.57–12.90)**
C/8-CATT	1 (0.3)	0 (0)	0.988	>999 (<0.001 - >999)
Total	356 (100)	160 (100)		

No deviation from Hardy–Weinberg equilibrium was detected in patients with atopic dermatitis or in the control subjects for either the *MIF –*173 single nucleotide polymorphism (*p* = 0.16; 1.0) or the CATT repeat element (*p* = 1.0; 0.17).

^a^ *P*-values for logistic regression analyses of the co-dominant models after controlling for sex and age, with log [total IgE] levels as a covariate. Significant associations are shown in bold.

In addition, the *MIF* C/5-CATT and the *MIF* C/7-CATT haplotypes were significantly associated with AD; the OR for the *MIF* C/5-CATT haplotype of the *MIF* −173 G/C and −794 CATT repeat polymorphisms was 9.7 (compared to the *MIF* G/5-CATT haplotype, 95% CI, 1.78–52.8, *p* = 0.009), and the OR for the *MIF* C/7-CATT haplotype of the *MIF* −173 G/C and −794 CATT repeat polymorphisms was 4.5 (compared to the G/5-CATT haplotype, 95% CI, 1.57–12.90, *p* = 0.005) (Table 3).

A multiple regression analysis with the *MIF* polymorphisms was performed for age and sex-adjusted log [total IgE] levels in the AD patients. The log [total IgE] levels were highly associated with age and sex among AD patients with the *MIF* −794 7-CATT polymorphism (*p* = 0.043) and the *MIF* C/7-CATT haplotype (*p* = 0.036) (Table 4). The initial analysis of the *MIF* −794 7-CATT polymorphism showed an association with a decrease in log [total IgE] levels in AD patients (*p* = 0.043), whereas the other loci showed no significant associations. The *MIF* C/7-CATT haplotype showed a dose-dependent effect on log [total IgE] levels in the haplotype analysis. The frequencies of the *MIF* C/7-CATT haplotype-containing genotypes decreased in order of IgE levels (*p* = 0.036), with significant effects in all three alternative models (co-dominant, dominant, and recessive).

**Table 4:** Regression analysis for age- and sex-adjusted log [total IgE] with *MIF* polymorphisms among patients with atopic dermatitis.

Locus	Genotype			*P*
-173 G>C	GG	GC	CC	0.464
	117 (5.62 ± 1.48)	51 (5.42 ± 1.96)	10 (4.91 ± 1.68)	
-794 5-CATT	-/-	-/+	+/+	0.300
	58 (5.71 ± 1.709)	88 (5.45 ± 1.58)	32 (5.35 ± 1.71)	
**-794 7-CATT**	**-/-**	**-/+**	**+/+**	**0.043**
	**132 (5.52 ± 1.56)**	**42 (5.70 ± 1.85)**	**4 (3.69 ± 0.98)**	
G/5-CATT	-/-	-/+	+/+	0.389
	63 (5.63 ± 1.69)	91 (5.41 ± 1.65)	24 (5.66 ± 1.48)	
G/6-CATT	-/-	-/+	+/+	0.518
	66 (5.33 ± 1.71)	94 (5.63 ± 1.63)	18 (5.65 ± 1.41)	
G/7-CATT	-/-	-/+	+/+	0.511
	163 (5.48 ± 1.65)	14 (6.05 ± 1.54)	1 (4.63)	
C/5-CATT	-/-	-/+	+/+	0.071
	165 (5.60 ± 1.61)	13 (4.52 ± 1.80)	UD	
C/6-CATT	-/-	-/+	+/+	0.282
	156 (5.46 ± 1.66)	21 (5.93 ± 1.47)	1 (6.91)	
**C/7-CATT**	**-/-**	**-/+**	**+/+**	**0.036**
	**146 (5.57 ± 1.55)**	**30 (5.42 ± 1.96)**	**2 (3.09 ± 1.08)**	
C/8-CATT	-/-	-/+	+/+	0.070
	177 (5.54 ± 1.63)	1 (2.41)	UD	

Genotype and haplotype distributions, means, standard deviations (SD) of log [total IgE] and *P*-values (F-test about source significance) are shown for the multiple regression analysis of log [total IgE] with each locus type adjusted for age and sex. Log [total IgE] levels were significantly associated with age and sex in patients with atopic dermatitis (*P* = 0.0312). UD: undetectable.

Age and sex-adjusted log [MIF] levels were not associated with the *MIF* polymorphisms in AD patients (*p* = 0.1841) in a multiple regression analysis (Table 5). However, the *MIF* C/5-CATT haplotype showed a dose-dependent effect on log [MIF] levels. The frequencies of the *MIF* C/5-CATT haplotype-containing genotypes decreased in order of MIF levels (*p* = 0.004), with significant effects in all three alternative models (co-dominant, dominant, and recessive).

**Table 5:** Regression analysis for age- and sex-adjusted log [MIF] levels with the *MIF* polymorphisms in patients with atopic dermatitis.

Locus	Genotype			*P*
-173 G>C	GG	GC	CC	0.182
	117 (6.03 ± 1.44)	51 (5.60 ± 1.61)	10 (5.43 ± 2.55)	
-794 5-CATT	-/-	-/+	+/+	0.803
	58 (5.7 ± 1.55)	88 (5.97 ± 1.61)	32 (5.81 ± 1.57)	
-794 7-CATT	-/-	-/+	+/+	0.921
	132 (5.86 ± 1.59)	42 (5.95 ± 1.62)	4 (5.66 ± 0.16)	
G/5-CATT	-/-	-/+	+/+	0.314
	63 (5.62 ± 1.67)	91 (6.01 ± 1.55)	24 (6.05 ± 1.42)	
G/6-CATT	-/-	-/+	+/+	0.983
	66 (5.87 ± 1.57)	94 (5.89 ± 1.68)	18 (5.85 ± 0.98)	
G/7-CATT	-/-	-/+	+/+	0.906
	163 (5.86 ± 1.61)	14 (6.07 ± 0.18)	1 (5.81)	
**C/5-CATT**	**-/-**	**-/+**	**+/+**	**0.004**
	**165 (5.97 ± 1.50)**	**13 (4.66 ± 2.02)**	**UD**	
C/6-CATT	-/-	-/+	+/+	0.090
	156 (5.89 ± 1.58)	21 (5.61 ± 1.48)	1 (8.82)	
C/7-CATT	-/-	-/+	+/+	0.922
	146 (5.88 ± 1.56)	30 (5.86 ± 1.75)	2 (5.62 ± 0.20)	
C/8-CATT	-/-	-/+	+/+	UD
	177 (5.91±1.52)	1 (0)	UD	

Genotype and haplotype distributions, means, standard deviations (SD) of log [MIF] and *P*-values (F-test about source significance) are shown for the multiple regression analysis of log [MIF] with each locus type adjusted for age and sex. Log [MIF] levels were not associated with age or sex in patients with atopic dermatitis (*P* = 0.1814). UD: undetectable.

## Discussion

We investigated the relationships between human *MIF* promoter polymorphisms and plasma log [total IgE] levels in Korean AD patients. Our data showed that *MIF* promoter polymorphisms in the −173 C allele and the *MIF* C/5-CATT and *MIF* C/7-CATT haplotypes were significantly associated with an increased risk for AD (Tables 2 and 3). In addition, the *MIF* C/7-CATT haplotype was also associated with a decrease in plasma log [total IgE] levels in a gene dose-dependent manner (Table 4).

The –173 G to C single nucleotide polymorphism (SNP) in the *MIF* gene was first identified by Donn *et al*. [24] in 2001, and is likely to confer susceptibility to juvenile idiopathic arthritis. Patients with juvenile idiopathic arthritis and the *MIF* –173 C SNP have increased blood and synovial fluids MIF levels, which were predictive of a shorter duration of clinical response to corticosteroid therapy [9, 25]. The –173 C promoter is more active than the –173 G promoter in CEMC7A cells, whereas, in A549 cells, the –173 G promoter is more active, suggesting that the −173 SNP may differentially affect promoter activity according to cell type. In addition, the *MIF* –173 G/C SNP has been associated with increased susceptibility to, or severity of, psoriasis, asthma, psoriatic arthritis, and AD. Wu *et al*. [13, 15] reported that the −173 C allele is associated with an increased risk for psoriasis in males, and late-onset psoriasis and childhood asthma in the Han population in northeastern China. Moreover, another study suggested association of the −173 C allele with susceptibility to psoriatic arthritis in a Mexican-Mestizo population [16]. Ma *et al*. [10] recently reported significant association between the *MIF* −173 G/C polymorphism and AD, and the CC genotype was significantly more frequent in the AD subgroup with rhinitis and/or asthma.

The *MIF* C/5-CATT and the *MIF* C/7-CATT haplotypes were significantly associated with an increased risk for AD in our study (Table 3). Although log [MIF] levels were not associated with age or sex among AD patients, the *MIF* C/5-CATT haplotype showed a dose-dependent effect on log [MIF] levels. The frequencies of the *MIF* C/5-CATT haplotypes decreased in order of MIF levels (*p* = 0.004), with significant effects in all three alternative models (Table 5). Mizue *et al*. [26] found a significant association between mild asthma and the *MIF* 5-CATT allele. However, they did not observe an association between other *MIF* alleles and asthma incidence [12]. The 5-CATT allele is associated with lower basal MIF promoter activity *in vitro* [11] and the homozygous 5-CATT allele is associated with F508del cystic fibrosis [27]. Recently, the combined effect of the −794 CATT and the −173 SNPs was reported in a patient with arthritis [28]. A case and control study performed in Japanese patients confirmed the association between CATT and −173 promoter polymorphisms in patients with atopy but not in those with asthma [12]. The risk of atopy was reduced in carriers of the −173 G/5-CATT haplotype, whereas it was increased in carriers of the −173 C/7-CATT haplotype. However, in A549 lung epithelial cells, the −173 G/7-CATT 5-CATT and C/6-CATT promoters exhibited lower activities than the −173 G/5-CAAT or 6-CAAT promoter [12]. The *MIF* C/7-CATT haplotype is also associated with asthma [13], juvenile idiopathic arthritis [9], rheumatoid arthritis [11], systemic lupus erythematosus [29], and skin diseases, such as psoriasis [14] and extensive alopecia areata [30].

We found that log [total IgE] levels were negatively associated with age and sex in AD patients, and with the *MIF* −794 7-CATT polymorphism (*p* = 0.043) and the *MIF* C/7-CATT haplotype (*p* = 0.036), in a gene dose-dependent manner (Table 4). The other loci showed no associations. Although the mechanism underlying the decrease in IgE levels in patients with AD remains unclear, polymorphisms in several cytokine genes, such as interleukin (IL)-3, IL-4, IL-5, IL-9, IL-13, and granulocyte-macrophage colony-stimulating factor (GM-CSF), regulate total serum IgE levels and are associated with atopy-related traits [31]. Our previous study suggested that the inhibition of innate immunity due to increased IL-10 production in subjects with *IL-10 ht2* [A-C-C-T] may be associated with decreased total serum IgE levels in AD patients [32].

*MIF* also codes for glycosylation inhibiting factor [33], which is an immunosuppressive cytokine involved in regulating antigen-specific IgE responses [34]. In our previous study, plasma MIF levels were significantly correlated with *Dp* and log [total IgE] levels, and *Df* was strongly correlated with MIF release in patients with AD [19]. Therefore, it is possible that the elevated total and specific IgE levels in patients with extrinsic AD reflect immediate hypersensitivity to *Df* and has considerable antigenic cross-reactivity.

The functional polymorphisms in the MIF gene promoter region are a causal factor for AD or antigen-specific IgE responsiveness, and they play regulatory roles in antigen-specific immune responses. However, previous studies with the mice genetically deficient in MIF showed conflicting results in IgE concentrations. Ovalbumin (OVA)-primed and inhalation-challenged asthma model mice with MIF deficiency had a reduction in the total and OVA-specific IgE [26], whereas MIF^−/−^ mice infected with *Schistosoma mansoni* [35] or *Taenia crassiceps* [36] produced normal amounts of Th_2_ cytokines and IgE.

Approximately 80% of AD patients that are called “extrinsic” AD react to allergens based on elevated serum IgE or show immediate skin test reactivity, whereas 20% have normal IgE levels and are not sensitized to environmental allergens [21, 37]. These “intrinsic” AD patients display a lower incidence of atopic march and filaggrin mutations compared with those with extrinsic AD [21, 38], although recent studies suggest that these patients may be sensitive to uncommon antigens that are not assessed on standard panels, such as metal or microbial antigens [21]. Our data suggest that intrinsic AD is associated with the effects of the −794 7-CATT locus and the *MIF* C/7-CATT haplotype on decreased total plasma IgE levels in AD patients, which also might be a marker for intrinsic AD.

In conclusion, MIF levels increased significantly in Korean patients with AD, and functional gene variants in the *MIF* promoter region were associated with total plasma IgE levels, which is similar to results in patients with chronic inflammatory skin disease. In particular, intrinsic AD was associated with the effects of −794 locus and the *MIF* C/7-CATT haplotype on decreased total plasma IgE levels.
